# WHOLE HEALTH(Y) AGING WITH GEROFIT: THE DEVELOPMENT OF A PILOT WELLNESS PROGRAM FOR OLDER VETERANS

**DOI:** 10.1093/geroni/igad104.2970

**Published:** 2023-12-21

**Authors:** Elizabeth Parker, Heidi Ortmeyer, Jamie Giffuni, Odessa Addison, Katherine Hall

**Affiliations:** UMSOM, Baltimore, Maryland, United States; VA, Baltimore, Maryland, United States; Baltimore VA, Baltimore, Maryland, United States; University of Maryland School of Medicine, Baltimore, Maryland, United States; Duke University/Veterans Health Administration, Durham, North Carolina, United States

## Abstract

The Veterans Health Administration (VHA) services over 9 million Veterans; approximately half are older than 65 years of age. Veterans are at a higher risk of comorbidities, including obesity and related chronic diseases, which contributes to higher health care costs, mobility disability, poor quality of life and even death. Gerofit is a VA Best Practice and Whole Health clinical exercise program for Veterans >65yrs that has positively impacted physical functioning, mental health symptoms, medication burden and 10-year mortality. This project describes the development of a comprehensive wellness and health promotion program incorporated into Gerofit, called Whole Healthy Aging Gerofit (WHAG). This novel program implemented wellness components to supplement the Gerofit in-person or virtual exercise options, including health education seminars, mindfulness classes, yoga classes, nutrition education classes, produce pick up service, and the Companion Dog Fostering program. WHAG deliverables included class attendance, individual Personal Health Inventories, observed physical function, self-reported physical activity levels, diet quality, perceived mental health, and Veteran satisfaction. The average Gerofit participant is approximately 73 years old, with obesity and multiple comorbid conditions (n=16), takes 10 medications, and is functionally impaired as assessed by gait speed (1.04 m/s). Programs that target healthy aging are needed to ensure the health of the population as it ages. This pilot program, WHAG, is one example of a comprehensive, patient centered wellness program that supports participants in setting their own goals and definitions of wellness to promote healthy aging.

